# A Randomised Clinical Study to Evaluate Efficacy on Gingival Health of 62% and 67% Sodium Bicarbonate Toothpastes

**DOI:** 10.3290/j.ohpd.b2403125

**Published:** 2021-12-08

**Authors:** Stephen Mason, Pranati Patil, Vaishali Karad

**Affiliations:** a Medical Affairs Director, GSK Consumer Healthcare, St. George’s Avenue, Weybridge, UK. Study design and interpretation; manuscript preparation and final approval to the content.; b Director, Smile Care Clinic Pvt., 1C 3-3, 2 Sujata Niwas, SV Road, Bandra (W), Mumbai, India. Conducting the study; manuscript preparation and final approval to the content.; c Orthodontist, Smile Care Clinic Pvt., 1C 3-3, 2 Sujata Niwas, SV Road, Bandra (W), Mumbai, India. Conducting the study; manuscript preparation and final approval to the content.

**Keywords:** dental plaque, gingivitis, gingival bleeding, sodium bicarbonate

## Abstract

**Purpose:**

This randomised, examiner-blind, parallel study compared gingival bleeding and plaque control following 12 weeks’ twice-daily use of 67%, 62% or 0% sodium bicarbonate (NaHCO_3_)-containing toothpastes.

**Materials and Methods:**

Adults with mild-to-moderate gingivitis, ≥ 20 gingival bleeding sites and bleeding after brushing were randomised to toothpastes containing 923 ppm sodium fluoride and either 67% NaHCO_3_, 62% NaHCO_3_ + 5% w/w silica, or 0% NaHCO_3_. Gingival bleeding was assessed with the Saxton and van der Ouderaa Bleeding Index (BI), plaque was assessed with the Turesky modification of the Quigley-Hein Plaque Index (TPI).

**Results:**

There were significantly fewer bleeding sites at Week 12 (primary endpoint) for 67% NaHCO_3_ (n = 110) and 62% NaHCO_3_ (n = 110) groups compared with the 0% NaHCO_3_ group (n = 110) (treatment differences: –3.1 [97.5% confidence interval (CI) –5.5, –0.7] P = 0.0068 and –2.4 [–4.8, 0.0] P = 0.0448, respectively). Statistically significant differences were also seen at Week 6 (P = 0.0361 for 67% NaHCO_3_, P = 0.0044 for 62% NaHCO_3_ compared to 0% NaHCO_3_). Compared with the 0% NaHCO_3_ group, BI was significantly lower in the 67% NaHCO_3_ group at Weeks 6 and 12 (P = 0.0477 and P = 0.0066, respectively). TPI was significantly lower in the 67% NaHCO_3_ and 62% NaHCO_3_ groups at Week 6 (P = 0.0336 and 0.0009, respectively) but not at Week 12. No statistically significant differences were found between the 67% NaHCO_3_ and 62% NaHCO_3_ toothpastes for any variable. No treatment-related adverse events were reported.

**Conclusion:**

Twice-daily brushing over 12 weeks with toothpastes containing 67% NaHCO_3_ or 62% NaHCO_3_ significantly improved plaque control and reduced indices of bleeding in participants with mild-to-moderate gingivitis.

Gum inflammation and bleeding upon stimulation, such as toothbrushing, are consumer-recognisable signs of gingivitis that may alert a person to a potential periodontal problem. If left untreated, gingivitis can progress to periodontitis, involving soft tissue damage, alveolar bone loss and loosening/loss of teeth.^11^ The role of accumulated plaque biofilm around the gingival margin is accepted as part of the aetiology of gingivitis.^11^ As plaque mass on tooth surfaces adjacent to gingival tissue increases, inflammation develops and flora composition shifts to one that produces proteolytic enzymes that cause further gingival damage.^16,23^ Good oral hygiene, including regular brushing with a toothpaste and interdental cleaning, is imperative in controlling plaque build-up and preventing resultant gum disease.^6,12,41,42^

This current study utilised an urban Indian population. A recent survey in a similar population found prevalence of gingivitis to be 54.2% and of chronic periodontitis to be 42.3%.^8^ Other oral health surveys in similar populations have found mild-to-moderate levels of plaque and gingival index scores, the latter of which correlated negatively with indices of oral health-related quality of life.^1,36^ However, one examination of general dental patients in India found that while many exhibited a high gingival index score on examination, a large percentage did not rate themselves as having gingivitis.^26^ While many studies recruit participants based on clinical indications of gingivitis such as gum redness and swelling, this current study used a positive response to a bleeding provocation at screening as the key recruitment criteria, as bleeding on brushing may be one of the key occurrences that alerts a person to the presence of gingivitis.^12^

Clinical studies performed in the late 1980s and early 1990s indicated that extended daily use of sodium bicarbonate (NaHCO_3_)/baking soda in fluoride toothpaste formulations could reduce gingival bleeding associated with pre-existing gingivitis.^32,33,43,44^ This was confirmed in a recent meta-analysis of nine studies where bleeding on provocation was assessed.^40^ A growing body of evidence also suggests that NaHCO_3_ enhances plaque removal.^29^ Single brushing studies and a meta-analysis of such have found that NaHCO_3_ toothpastes can remove significantly more plaque than non-NaHCO_3_ toothpastes,^10,24,40^ including those with high-cleaning abrasive systems such as hydrated silica and dicalcium phosphate^15^ or those containing antimicrobial ingredients.^29^ The latter paper detailed analysis comparing different levels of NaHCO_3_, from 20% to 65%, and found a positive relationship between NaHCO_3_ concentration and enhanced plaque removal by brushing.^29^ These results have been confirmed by longer studies comparing NaHCO_3_ toothpastes to non-NaHCO_3_ toothpastes^3,14,35^ including those with an antimicrobial system^10^ or calcium carbonate control toothpaste.^44^

While the mechanism of action of NaHCO_3_ has not been fully established, it has been hypothesised to relate to (i) the physical displacement of plaque by NaHCO_3_ crystals; (ii) a NaHCO_3_-induced reduction in the viscosity of the polysaccharide matrix of plaque making it easier to brush away; or (iii) a NaHCO_3_-induced reduction in the bond strength between plaque bacteria and the tooth surface.^27,29^ However, there are relatively few clinical studies using modern methods that confirm the long-term effects of NaHCO_3_-containing toothpastes and support the need to postulate hypotheses for its mode of action.

The purpose of this study was to evaluate and compare the effects of twice-daily brushing with toothpastes containing either 67% NaHCO_3_, 62% NaHCO_3_ plus 5% w/w silica or 0% NaHCO_3_ plus 14% w/w silica silica (all with 923 ppm fluoride, the cosmetic fluoride level permitted in India, as sodium fluoride [NaF]) after 6 and 12 weeks on gingival health and plaque control in a population with mild-to-moderate gingivitis.^5^ Of note, this study used a ‘dirty mouth’ design,^15,33,39,44,45^ wherein there is no prophylaxis prior to the use of study toothpastes. This is in contrast to many previous studies, including those by the study sponsor, of a similar 67% NaHCO_3_ (1,100 ppm fluoride as NaF) toothpaste, where a prophylaxis was carried out.^3,18^ This is hoped to provide a wider picture of the efficacy of NaHCO_3_ within a toothpaste and reflect a more ‘real-world situation’ where a consumer uses a toothpaste purchased from the supermarket and has not had a prior dental prophylaxis.

## Materials and Methods

This was a randomised, examiner-blind, three-treatment, parallel-group, stratified (by baseline number of bleeding sites and smoking status) study conducted at a clinical research facility in India. The study protocol was reviewed and approved by the centre’s Institutional Ethics Committee (Registration number ECR/463/Inst/MH/2013) and was performed in accordance with the requirements specified in the Declaration of Helsinki and relevant local laws and regulations. All eligible participants provided written informed consent before initiation of study procedures. There was one amendment to the protocol, a correction of a typographical error, that had no direct effect on study process or outcomes. Anonymised individual participant data and study documents can be requested for further research from www.clinicalstudydatarequest.com.

### Participants

Eligible participants were aged ≥18 years and were in good general and oral health. At the screening visit, participants were required to have ≥20 permanent gradable teeth with mild-to-moderate gingivitis (as assessed by the investigator VK) and a positive response to bleeding after supervised brushing with a standard toothpaste and toothbrush (evidence of blood in the expectorant or bleeding on brushing). Participants were excluded from participation if they were pregnant; breastfeeding or required prophylactic antibiotic treatment prior to dental therapy. They were also excluded if they had: current active caries; excessive calculus; more than three periodontal pockets measuring ≥5 mm in depth; other severe oral/gingival conditions or other medical conditions affecting gingival bleeding; restorations in a poor state of repair or orthodontic appliances; a known/suspected intolerance/hypersensitivity to the study material; used chewing tobacco, paan (betel leaf), paan-masala, gutkha or other chewing tobacco products within 6 months of screening. The use of antibiotics or systemic medication that could affect gingival conditions was not permitted in the 2 weeks prior to screening or throughout the study.^21^

### Study Procedures

At screening, participants were issued with a washout toothpaste containing 0% NaHCO_3_ plus 923 ppm sodium fluoride (NaF) and an Aquafresh Clean Control toothbrush (GSK Consumer Healthcare [GSKCH], Weybridge, UK) for use as normal until the baseline visit, scheduled for 7–14 days after screening. They were instructed to abstain from brushing for 12 h (+5 h, –2 h) prior to the baseline visit. At baseline, participants underwent a Bleeding Index assessment, with the number of bleeding sites derived from the Saxton and van der Ouderaa Bleeding Index^34^ (see below for details), carried out by VK here and at all timepoints.

Participants were stratified according to baseline number of bleeding sites (low [<45] or high [≥45]) and smoking status (yes or no, as smoking may affect gum health) and then randomised to a group according to a computer-generated schedule provided by the Biostatistics Department of the study sponsor in a 1:1:1 allocation ratio using a block size of six. Groups were assigned the following toothpastes, all of which contained purified water, glycerol, xanthan gum, saccharin sodium and flavouring. Additional ingredients for each toothpaste were listed as follows:

- (i) 67% NaHCO_3_ plus 923 ppm NaF (67% NaHCO_3_ group), also including cocamidopropyl betaine and colouring;
- (ii) 62% NaHCO_3_ plus 923 ppm NaF and 5% w/w silica (62% NaHCO_3_), also including cocamidopropyl betaine and titanium dioxide;
- (iii) 0% NaHCO_3_ plus 923 ppm NaF plus 14% w/w silica (0% NaHCO_3_ group), also including sorbitol, polyethylene glycol, sodium lauryl sulphate (SLS), carrageenan, sodium hydroxide and titanium dioxide.

Participants were instructed to brush their teeth with a strip of toothpaste covering the entire head of the provided toothbrush (approximately 1.5 g) for 1 timed minute, twice a day for 12 weeks. Study toothpastes were supplied in plain white tubes with a study label affixed to each tube. The examiner, study statistician and other employees of the sponsor who may have influenced study outcomes were blinded to treatment allocation.

### Assessments

Participants underwent full dental plaque oral soft tissue (OST) examination (carried out by PP) and gingival bleeding assessments at baseline, Week 6 and Week 12 visits (carried out by VK). Gingival bleeding was assessed according to the Saxton and van der Ouderaa Bleeding Index (BI),^34^ performed by a single trained examiner (PP) using a colour-coded periodontal probe engaged approximately 1 mm into the gingival crevice. A moderate pressure was used while sweeping from interproximal to interproximal along the sulcular epithelium. For each tooth, both facial and lingual sides were assessed. The BI scoring system was as follows: 0 (no bleeding after 30 s), 1 (bleeding upon probing after 30 sec) or 2 (immediate bleeding upon probing). A site was considered to be bleeding if the score was 1 or 2; overall BI was calculated as mean score across all tooth sites.

Plaque was assessed by PP after disclosing using 5 ml of Red Cote (Sunstar Americas, Schaumburg, IL, US), swilled for 10 s then expectorated, as per label instructions. Assessment was carried out at six sites per tooth (mesiofacial, facial, distofacial, mesiolingual, lingual and distolingual surfaces) according to the Turesky modification of the Quigley-Hein Plaque Index (TPI),^30,38^ with each tooth scored from 0 (no plaque) to 5 (plaque covering 2/3 or more of the crown of the tooth). The overall TPI included all surface scores and was calculated taking the average over all tooth sites for a participant; the Interproximal TPI (ITPI) was limited to the mesiofacial, distofacial, mesiolingual, and distolingual surfaces and was calculated taking the average over these sites only for a participant. To assess repeatability, the examiner completed one repeat plaque assessment, at least 10 min after the first, on two participants each day plaque assessments were performed.

Spontaneously reported adverse events (AEs) and any abnormalities in the OST examination were recorded from the time of supervised brushing with the washout paste at the screening visit until 5 days after the last administration of study product. The investigator assessed the relationship between investigational product and the occurrence of each AE using clinical judgment and graded the AE as mild, moderate or severe. Treatment-emergent AEs were reported for the safety population, which included all randomised participants who received the study treatment.

### Statistical Methods

Approximately 600 participants were to be screened in order to randomise 110 participants per treatment group (approximately 330 participants in total), to ensure at least 100 participants per treatment group completed the study. A sample size of 100 participants per treatment group was calculated to have 85% power to detect a mean treatment difference between groups of 20% (difference of 5.8 in the number of bleeding sites with a within-group standard deviation of 12.5) with a two-sided statistical significance level of 0.05, including Dunnett’s adjustment for the comparison of two experimental treatments versus a control.

The efficacy analysis was performed on a modified intent-to-treat (mITT) population, defined as all participants who received the study treatment and had at least one post-baseline efficacy measurement.^3,18^ The primary efficacy variable was number of bleeding sites at Week 12 with the primary comparison being between each of the two experimental toothpastes (67% NaHC0_3_ and 62% NaHC0_3_) and the reference toothpaste (0% NaHC0_3_). These comparisons were carried out with a mixed model repeated measures (MMRM) analysis that ensured that participants with missing responses at Week 12 were incorporated into the statistical analysis provided they completed the Week 6 assessment. The statistical model included factors for treatment group, time, smoking status and treatment time interaction, with the baseline number of bleeding sites as a covariate, and the baseline time interaction term. The unit of analysis was of each participant and an unstructured covariance matrix was specified. For all analyses, the observed margin option in the analysis program (SAS Studio Version 9.4; SAS Institute, Cary, NC, USA) was used when estimating least square means. For the primary comparison, an adjustment was made to the statistical significance level using a Bonferroni correction for two treatment comparisons. Hence, hypothesis tests were performed at an adjusted statistical significance level of 2.5% and differences in adjusted means was presented together with an adjusted confidence interval (CI) of 97.5%.

The Bonferroni method of adjustment was a change to the methodology originally proposed, Dunnett’s adjustment. While conducting the primary efficacy analysis, it became evident that the more conservative Bonferroni correction would be more appropriate in a repeated measures framework. Assumptions of normality and homogeneity of variance were investigated and considered satisfied.

Secondary efficacy variables included number of bleeding sites at Week 6 and BI, TPI and ITPI at Weeks 6 and 12. Between-treatment comparisons were made using a MMRM analysis and the statistical model included factors for number of bleeding sites strata level (for the analysis of BI, TPI and ITPI), treatment group, time, smoking status and treatment time interaction, the corresponding baseline score as a covariate, and the corresponding baseline time interaction term. No adjustment was made to the statistical significance level of the analysis of any of the secondary parameters. Though not a defined objective, a planned subgroup analysis was performed on number of bleeding sites and BI according to baseline number of bleeding sites (low [<45] or high [≥45]). This analysis was performed using the same MMRM analysis as for the secondary efficacy variables, with the addition of the treatment time number of bleeding sites strata interaction term.

A post-hoc analysis was added after study un-blinding for percentage change from baseline in the number of bleeding sites. This was analysed using the same MMRM analysis as for the primary efficacy variable of number of bleeding sites.

A weighted Kappa coefficient was calculated to assess the intra-examiner reliability in terms of TPI scoring. Reliability was deemed to be excellent if Kappa was >0.75, fair to good if Kappa was ≤0.75 to ≥0.4, and poor if Kappa was <0.4.

## Results

**Fig 1:**
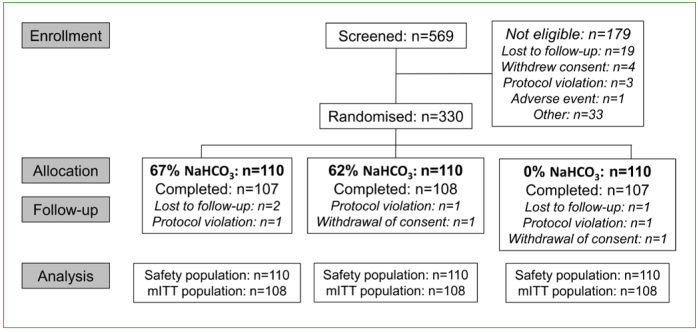
Participant disposition.

The first participant was enrolled into the study in May 2012 with the last participant completing the study in September 2012. Study flow is shown in Figure 1. Lost to follow-up or withdrawal of consent following randomisation meant that the participant was not included in any part of the study. Protocol violations only occurred on one study visit so data was only excluded at that timepoint. The 330 participants included in the safety population had a mean age of 21.3 years (range 18–41); slightly more were male (n = 177, 53.6%). There were no notable differences in baseline characteristics between treatment groups (Table 1).

**Table 1:** Participant demographics and baseline characteristics (safety population)

	67% NaHCO3 (n = 110)	62% NaHCO3 (n = 110)	0% NaHCO3 (n = 110)
**Sex, n (%)**			
Male	57 (51.8)	59 (53.6)	61 (55.5)
Female	53 (48.2)	51 (46.4)	49 (44.5)
**Mean age, years (±SD)**	21.1 (4.06)	21.3 (4.27)	21.5 (4.70)
**Range**	18–38	18–41	18–40
**Strata, n (%)**			
<45 bleeding sites, non-smoker	38 (34.5)	40 (36.4)	39 (35.5)
<45 bleeding sites, smoker	22 (20.0)	22 (20.0)	21 (19.1)
≥45 bleeding sites, non-smoker	38 (34.5)	37 (33.6)	38 (34.5)
≥45 bleeding sites, smoker	12 (10.9)	11 (10.0)	12 (10.9)

### Efficacy

#### Gingival bleeding

**Fig 2:**
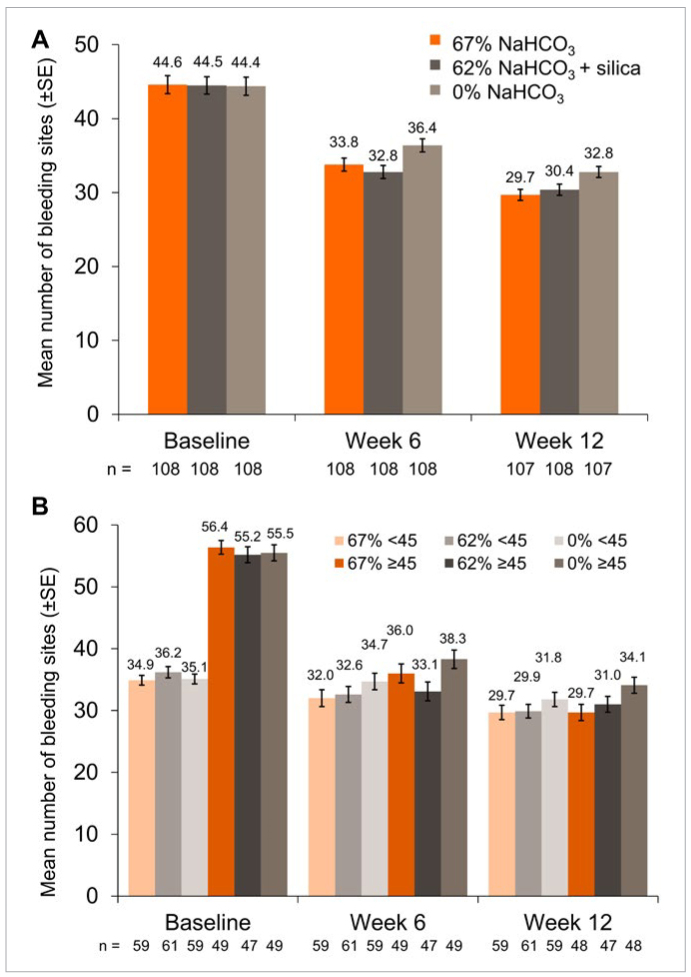
Mean number of bleeding sites (mITT population) in (A) each treatment group and (B) each treatment group according to baseline bleeding sites (<45 and ≥45 sites). Raw means are presented at baseline, adjusted means at Weeks 6 and 12.

The mean number of bleeding sites decreased significantly from baseline in each group at Weeks 6 and 12 (P <0.0001 for all) (Fig 2a). Compared with the 0% NaHCO_3_ group, there were statistically significantly fewer mean number of bleeding sites at both timepoints in participants in the 67% NaHCO_3_ group and 62% NaHCO_3_ group (Table 2). When assessed according to number of bleeding sites at baseline (Fig 2b, Table 2), for those with ≥45 bleeding sites there was a statistically significant difference in number of bleeding sites in the 67% NaHCO_3_ group versus the 0% NaHCO_3_ group at Week 12 and between the 62% NaHCO_3_ group versus the 0% NaHCO_3_ group at Week 6. No between-treatment differences were seen in the low bleeding site subgroup (<45) at either timepoint.

**Table 2:** Summary of between-treatment differences in mean number of bleeding sites (mITT population)

Comparison	Week	Difference1 (CI)2	% Diff3	*P* value	Bleeding sites subanalysis	Difference1 (95% CI)	% Diff3	*P* value
67% NaHCO3 vs 0% NaHCO3	6	–2.6 (–5.0, –0.2)	–7.2	0.0361	<45	–2.8 (–6.1, 0.5)	–8.0	0.0980
					≥45	–2.4 (–6.0, 1.3)	–6.1	0.2001
	12	–3.1 (–5.5, –0.7)^2^	–9.5	0.0068	<45	–2.1 (–4.9, 0.7)	–6.7	0.1403
					≥45	–4.3 (–7.4, –1.2)	–12.7	0.0064
62% NaHCO3 vs 0% NaHCO3	6	–3.5 (–6.0, –1.1)	–9.8	0.0044	<45	–2.1 (–5.4, 1.1)	–6.2	0.1975
					≥45	–5.2 (–8.9, –1.6)	–13.6	0.0053
	12	–2.4 (–4.8, 0.0)^2^	–7.4	0.0448	<45	–1.9 (–4.7, 0.9)	–5.9	0.1867
					≥45	–3.0 (–6.2, 0.1)	–8.9	0.0576
67% NaHCO3 vs 62% NaHCO3	6	0.9 (–1.5, 3.4)	2.9	0.4471	<45	–0.6 (–3.9, 2.6)	–1.9	0.7039
					≥45	2.9 (–0.8, 6.5)	8.6	0.1255
	12	–0.7 (–2.8, 1.4)	–2.3	0.5092	<45	–0.2 (–3.0, 2.6)	–0.8	0.8682
					≥45	–1.3 (–4.4, 1.8)	–4.2	0.4126

^1^ A negative difference favours first named treatment. Difference is adjusted mean. ^2^ CI of 97.5% to adjust for multiple comparisons for primary comparisons at Week 12 only; all other comparisons presented with 95% CI. ^3^ Percentage difference: second named treatment taken as reference for per cent difference calculation ([Diff/Ref] 100%).

**Fig 3:**
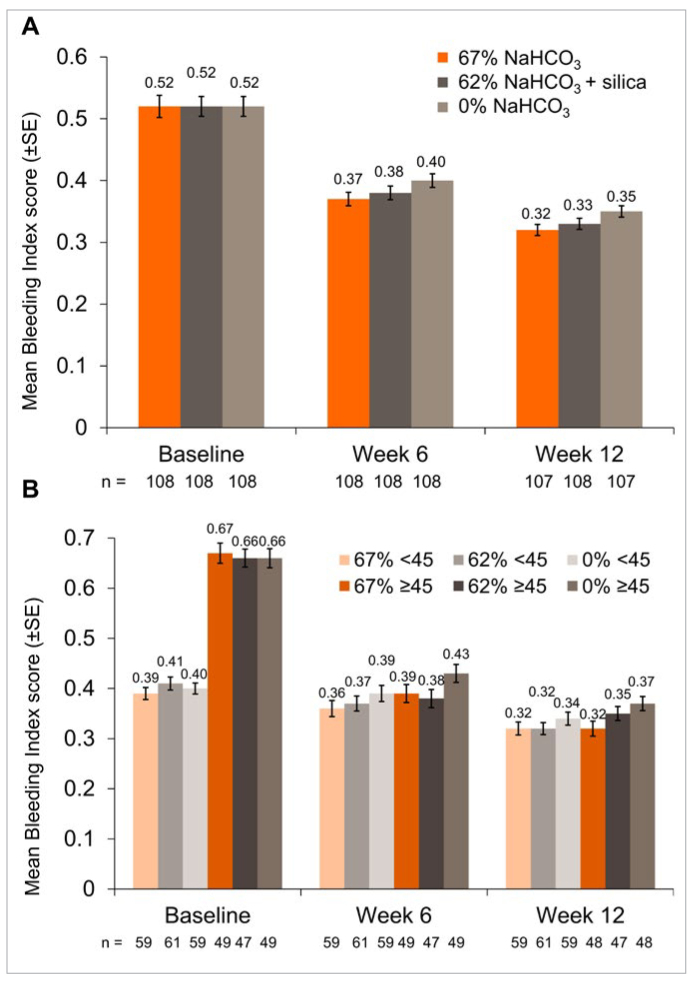
Mean Bleeding Index (mITT population) in (A) each treatment group and (B) each treatment group according to baseline bleeding sites (<45 and ≥45 sites). Bleeding Index was scored on a scale of 0 (no bleeding after 30 secs) to 2 (immediate bleeding observed). Overall Bleeding Index score was the average of all tooth sites. Raw means are presented at baseline, adjusted means at Weeks 6 and 12.

Mean BI decreased over the 12-week study in both NaHCO_3_ toothpaste groups compared to baseline (Fig 3a). The difference between the 67% NaHCO_3_ and 0% NaHCO_3_ groups was statistically significant at Weeks 6 and 12 (Table 3). Subgroup analysis showed that participants in the ≥45 bleeding site group using 67% NaHCO_3_ toothpaste had a statistically significant reduction in BI at Week 12 relative to the 0% NaHCO_3_ group and those using 62% NaHCO_3_ toothpaste showed a statistically significant reduction in BI at Week 6 relative to those treated with 0% NaHCO_3_ (Fig 3b; Table 3). No between-treatment differences were seen in the low bleeding site subgroup at either timepoint.

**Table 3:** Summary of between-treatment differences in mean bleeding index score (mITT population)

Comparison	Week	Difference1 (95% CI)	% Diff2	P value	Subanalysis by no. bleeding sites	Difference1 (95% CI)	% Diff2	P value
67% NaHCO3 vs 0% NaHCO3	6	–0.03 (–0.06, 0.00)	–7.4	0.0477	<45	–0.02 (–0.06, 0.02)	–6.0	0.2581
					≥45	–0.04 (–0.08, 0.01)	–8.9	0.0900
	12	–0.03 (–0.06, –0.01)	–9.4	0.0066	<45	–0.02 (–0.05, 0.01)	–5.9	0.2310
					≥45	–0.05 (–0.09, –0.01)	–13.3	0.0064
62% NaHCO3 vs 0% NaHCO3	6	–0.03 (–0.06, 0.00)	–7.2	0.0525	<45	–0.02 (–0.06, 0.02)	–4.2	0.4186
					≥45	–0.05 (–0.09, 0.00)	–10.6	0.0460
	12	–0.02 (–0.04, 0.00)	–5.9	0.0899	<45	–0.01 (–0.05, 0.02)	–4.4	0.3676
					≥45	–0.03 (–0.06, 0.01)	–7.4	0.1284
67% NaHCO3 vs 62% NaHCO3	6	0.00 (–0.03, 0.03)	–0.2	0.9665	<45	–0.01 (–0.05, 0.03)	–1.8	0.7396
					≥45	0.01 (–0.04, 0.05)	1.9	0.7494
	12	–0.01 (–0.04, 0.01)	–3.8	0.3002	<45	–0.01 (–0.04, 0.03)	–1.6	0.7583
					≥45	–0.02 (–0.06, 0.01)	–6.3	0.2319

^1^ A negative difference favours first named treatment. Difference is adjusted mean. ^2^ Percentage difference: second named treatment taken as reference for per cent difference calculation ([Diff/Ref] 100%).

No statistically significant differences between the 67% NaHCO_3_ and 62% NaHCO_3_ groups were reported in any analysis.

#### Plaque

**Fig 4:**
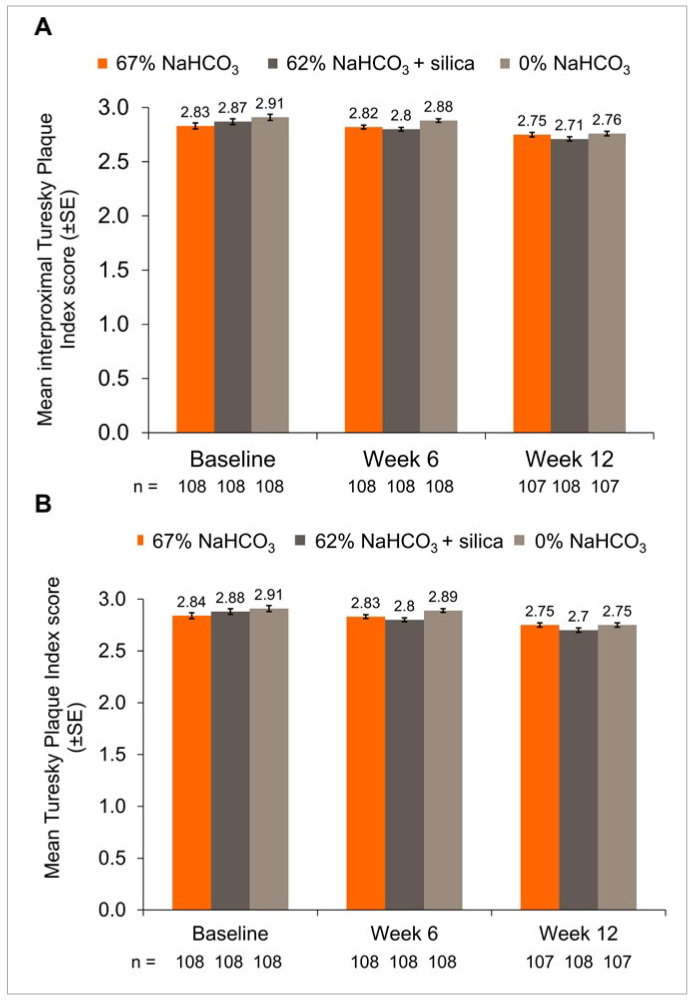
Mean (A) overall Turesky plaque index and (B) interproximal Turesky Plaque Index (mITT population). TPI was scored on a scale of 0 (no plaque) to 5 (plaque covering two-thirds or more of the crown of the tooth). Raw means are presented at baseline, adjusted means at Weeks 6 and 12.

Figure 4 demonstrates the TPI and ITPI scores at baseline, Week 6 and Week 12. At Week 6, there was statistically significantly lower overall plaque and interproximal plaque scores among participants who brushed with the 67% NaHCO_3_ and 62% NaHCO_3_ toothpastes compared with the 0% NaHCO_3_ toothpaste (Table 4). These differences were not statistically significant at Week 12. The repeatability analysis of the TPI (based on 81 participants) showed excellent agreement between the first and repeat assessment (κ = 0.882; 95% CI 0.877, 0.887).

**Table 4:** Summary of between-treatment differences in mean Plaque Index score (mITT population)

Comparison	Week	Overall TPI			Interproximal TPI		
		Difference1 (95% CI)	% Diff2	P value	Difference1 (95% CI)	% Diff2	P value
67% NaHCO3 vs 0% NaHCO3	6	–0.05 (–0.10, 0.00)	–1.9	0.0336	–0.06 (–0.11, –0.01)	–2.1	0.0151
	12	0.00 (–0.06, 0.06)	0.1	0.9118	–0.01 (–0.06, 0.05)	–0.3	0.7961
62% NaHCO3 vs 0% NaHCO3	6	–0.08 (–0.13, –0.03)	–2.9	0.0009	–0.09 (–0.13. –0.04)	–3.0	0.0005
	12	–0.04 (–0.10, 0.01)	–1.6	0.1422	–0.05 (–0.10, 0.01)	–1.7	0.1019
67% NaHCO3 vs 62% NaHCO3	6	0.03 (–0.02, 0.08)	1.1	0.2322	0.03 (–0.02, 0.07)	0.9	0.2877
	12	0.05 (–0.01, 0.10)	1.7	0.1149	0.04 (–0.02, 0.10)	1.5	0.1689

^1^ A negative difference favours first named treatment. Difference is adjusted mean. ^2^ Second named treatment taken as reference for percent difference calculation ([Diff/Ref]*100%).

#### Safety

In the 67% NaHCO_3_ group, six participants reported eight treatment-emergent AEs (TEAEs) of which three were oral (toothache, gingival ulceration, lip ulceration). In the 62% NaHCO_3_ group, six participants reported seven TEAEs of which six were oral (toothache [two], lip ulceration, gingival pain, glossodynia, lip exfoliation). In the 0% NaHCO_3_ group, nine participants reported 12 TEAEs, of which two were oral (gingival ulceration, pain in erupting third molar). None of the TEAEs were considered by the examiner to be treatment related. All TEAEs were mild and there were no serious AEs or withdrawal due to AEs reported during the study.

## Discussion

The use of NaHCO_3_ toothpastes have been shown to affect clinical outcomes associated with gingival health, such as gingivitis and gingival bleeding, when compared to baseline,^33,44,45^ to non-NaHCO_3_ toothpastes,^15,40^ and to toothpastes containing tartar-control ingredients such as calcium carbonate.^44^ This study examined the effect of different concentrations of NaHCO_3_ in toothpastes on participants with established gingivitis and bleeding on provocation. Other studies in Indian cohorts have investigated a variety of interventional measures with participants with very similar baseline gingivitis and plaque measurements as used in this study,^7,9,17,28^ confirming the methodology in this paper is relevant to the population.

In this study, those using the 67% NaHCO_3_ toothpaste demonstrated statistically significantly fewer bleeding sites and a lower BI compared to those using the 0% NaHCO_3_ toothpaste at both 6 and 12 weeks. Participants using the 62% NaHCO_3_ toothpaste also demonstrated statistically significantly fewer bleeding sites and numerically, though not significantly, a lower Bleeding Index compared to the 0% NaHCO_3_ toothpaste group. Subanalysis by number of bleeding sites indicated that statistically significant differences in bleeding site/Bleeding Index scores primarily occurred in those with ≥45 bleeding sites, suggesting a benefit of NaHCO_3_ particularly to people with higher levels of gingivitis.

It is of note that the study was powered to detect a 20% difference in the number of bleeding sites, as suggested by the American Dental Association as being suitable for assessment of gingivitis.^5^ At Week 12, the differences between the 0% NaHCO_3_ toothpaste and the 67% and 62% NaHCO_3_ toothpastes were 9.5% and 7.4% respectively, so while these were statistically significant, they are smaller than the effect size considered important enough to detect prior to the study start. That said, the study also demonstrated markedly less variability than predicted at outset, thus the reason for the statistical significance. As there was a reduction from baseline in all groups, there may have been a ‘Hawthorne’ effect whereby mere participation in the study led all participants to change their brushing behaviour to one more conducive to plaque removal.

There were no differences between the 67% and 62% toothpastes on any measures, which could suggest that 62% NaHCO_3_ is a threshold for the amount needed to produce an effect. However, previous studies have found statistically significant differences in BI scores with toothpastes containing 35% or 20% NaHCO_3_.^14,35^ A companion study, using the same toothpastes but including a prophylaxis prior to the study start, also found statistically significant bleeding index/bleeding site number differences at both 6 and 12 weeks but additionally had clinically meaningful percentages between NaHCO_3_ and non-NaHCO_3_ toothpastes.^19^ This confirms the validity of a combined approach to gingivitis management of both daily toothbrushing with a NaHCO_3_ toothpaste and regular clinic-based prophylaxis.

In this current study, the results for overall and interproximal plaque reduction are consistent with enhanced and early plaque removal. After 6 weeks of use, there was statistically significant less plaque (indicating greater plaque control benefit) among participants using either of the NaHCO_3_ toothpastes compared to the non-NaHCO_3_ toothpaste (P <0.05 for all). That this occurred both for overall and interdental plaque is reflected in a meta-analysis where NaHCO_3_ toothpastes were found to be better at removing plaque from interdental and lingual surfaces of the dentition.^37^ However, by 12 weeks the difference was not statistically significant.

Both the quantity and complexity of plaque influence clinical signs of inflammation.^20^ Depending on its age and thickness, the plaque matrix may be thin and porous or gelatinous and less porous.^31^ These factors may make aged plaque resistant to the effect of toothpaste ingredients and mechanical removal by a toothbrush. Old plaque left accumulated in sheltered areas of the mouth may contribute to faster plaque regrowth on accessible surfaces after brushing. Most gingival health improvement studies include a scaling and prophylaxis at baseline to bring plaque scores on tooth surfaces to near zero to represent expected standard of dental care. Where such standard of dental care is employed, the objective of plaque control then becomes maintenance of gingival health or a slower return of gingivitis over time and reflects an ‘ideal’ oral health program that commences with professional care and encourages daily toothbrushing.^6,41,42^ In this study, there was no prophylaxis before toothpaste use and comparisons were made between baseline plaque scores and after 6 and 12 weeks brushing. Although prevalent in the literature,^15,33,39,44,45^ this design is less frequently used, but is considered to reflect a more complex situation of a patient/consumer infrequently attending a dental office for a ‘scale and polish’. The companion study to this one^19^ did include a prophylaxis prior to 12 weeks’ brushing with the same toothpastes. Results there showed statistically significant differences in overall and interdental plaque scores for both toothpastes after 12 weeks’ use. Taken together, the results indicate the importance of combining a professional prophylaxis with at-home continual use of a plaque-controlling toothpaste for maintenance of gingival health.

One potential limitation of this study is that, while the investigators assessed the occurrence of plaque and mild–moderate gingivitis using their subjective expertise, it is common with many studies of this type in the literature. Another potential limitation was that the base formulations of the toothpaste were not identical due to the rheological need to produce a consumer acceptable toothpaste. It cannot be wholly discounted that the surfactant differences between cocamidopropyl betaine (in both NaHCO_3_ toothpastes) and SLS (in the 0% NaHCO_3_ toothpaste) affected bacterial growth differently; however, it is generally assumed that SLS is likely to have the most potent antibacterial effect and this was only present in the control toothpaste. Other small formulation differences, such as titanium dioxide^2^ in the 62% and 0% NaHCO_3_ toothpastes, and sodium hydroxide^45^ in the 0% NaHCO_3_ toothpaste, and other formulation differences including polyethylene glycol^4^ and carrageenan,^13^ either do not have any known antibacterial properties or, at the concentrations used, are not expected to impact antibacterial performance/gingival health. Similarly while the silica used in the 62% and 0% NaHCO_3_ toothpastes is an abrasive, capable of removing stained pellicle,^25^ as an ingredient it was considered the benchmark for plaque removal; therefore, the different performances of the toothpastes are assessed relatively.

## Conclusions

Twice-daily brushing with toothpaste containing 67% or 62% NaHCO_3_ significantly reduced the number of bleeding sites in participants with mild-to-moderate gingivitis compared to a regular toothpaste (0% NaHCO_3_). This study potentially confirms that high concentrations of NaHCO_3_-containing toothpastes are important adjuncts to gingival health improvement.
